# A 14-Year-Old Female With Several-Week History of Rash, Worsening Diffuse Arthralgias, and Fevers

**DOI:** 10.7759/cureus.27846

**Published:** 2022-08-10

**Authors:** Seth J Deskins, Felistia Crowder, Sydney Downey, Jacob Gelman, Richard Brant, Brian Peppers

**Affiliations:** 1 Internal Medicine-Pediatrics, West Virginia University Medicine, Morgantown, USA; 2 Pediatrics, West Virginia University, Morgantown, USA; 3 Internal Medicine-Pediatrics, West Virginia University, Morgantown, USA; 4 Allergy/Immunology, West Virginia University, Morgantown, USA

**Keywords:** pediatric immunology, pediatrics, case report, soluble il-2 receptor, systemic juvenile idiopathic arthritis, macrophage activation syndrome (mas)

## Abstract

Macrophage activation syndrome (MAS) as the initial presentation of systemic juvenile idiopathic arthritis (sJIA) is an uncommon and difficult diagnosis to ascertain. However, it remains critical to establish the diagnosis since MAS is a potentially life-threatening systemic inflammatory condition. Prompt recognition can lead to early initiation of treatment with corticosteroids and overall improved outcomes. Here, we present a case of a 14-year-old female with MAS as the initial manifestation of sJIA.

## Introduction

Macrophage activation syndrome (MAS) as the initial presentation of systemic juvenile idiopathic arthritis (sJIA) is a rare and difficult diagnosis to ascertain, and the final diagnosis can often be delayed [1,2]. MAS is a systemic inflammatory response that can be life-threatening progressing to multi-organ failure if unrecognized [2]. Prompt treatment should be initiated when workup is consistent with diagnosis typically with high-dose steroids [1]. If patients fail to respond to steroids, alternative disease-modifying agents should be explored [1]. Here, we present a young female whose diagnosis was delayed due to atypical presentation.

## Case presentation

**Figure 1:**
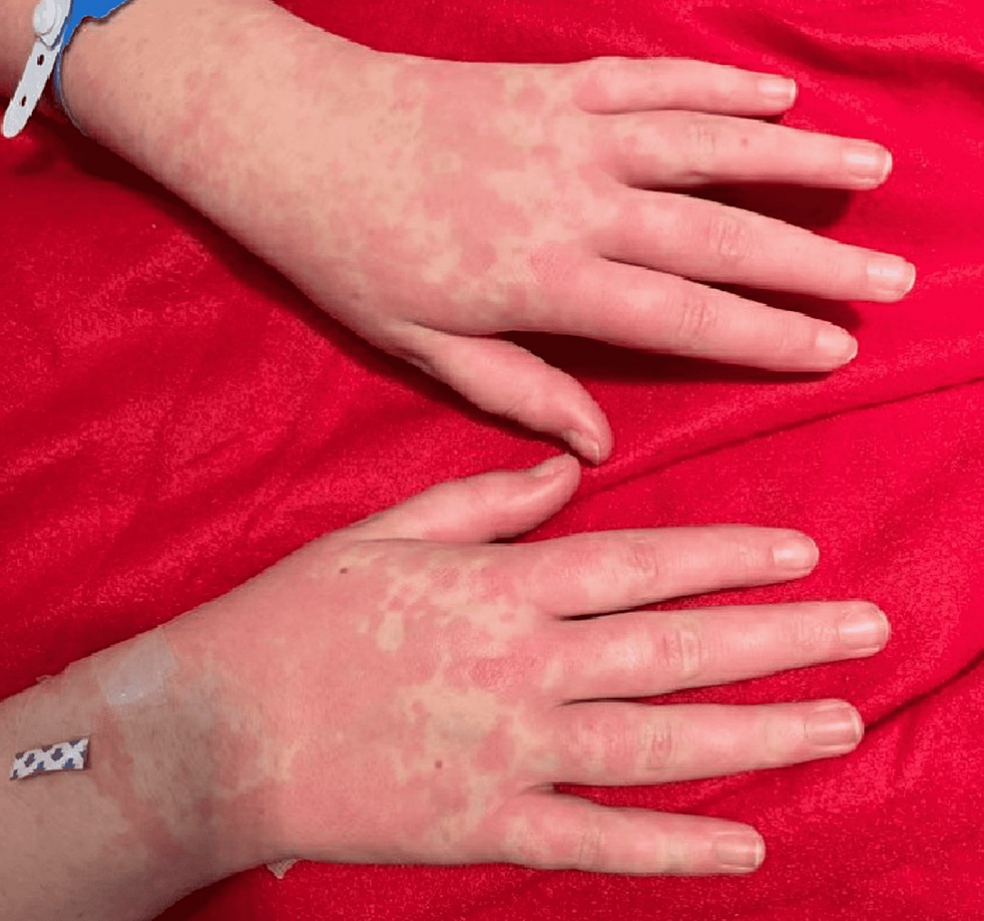
Rash present on the dorsum of hands

A previously healthy 14-year-old female was directly admitted to our institution with a prolonged course of recurrent rash, worsening diffuse arthralgias and myalgias, and intermittent daily fevers occurring for a couple of weeks. She was initially admitted two weeks prior due to a three-week history of generalized hives and worsening joint pains in the absence of fevers. Evaluation during the first admission was significant for an erythrocyte sedimentation rate (ESR) of 52 mm/hour, C-reactive protein (CRP) level of 82.5 mg/L, white blood cell (WBC) count of 16.9 × 10^3^/uL, and platelet level of 421 × 10^3^/uL. In addition, she yielded a negative respiratory viral PCR panel, negative blood and throat cultures, and an unremarkable chest X-ray. A diagnosis of acute urticaria and joint pain of unclear etiology was made, and she was discharged home on carbinoxamine, montelukast, famotidine, and a 10-day course of doxycycline for empiric Lyme disease treatment in addition to close follow-up with her primary care physician and allergist/immunologist. Due to the progression of her symptoms and the onset of daily fevers following the initial hospital discharge, she was readmitted. Upon readmission, her examination result is significant for hypotension (89/56 mmHg), tachycardia (112 beats/minute), and fever (101.3ºF) with a blanching, confluent maculopapular rash on the dorsum of both hands (Figure 1).

Her urticaria was no longer present. Initial laboratory analysis was remarkable for a leukocytosis of 15 × 10^3^/uL with neutrophilic predominance, a mildly prolonged prothrombin time (PT) of 21.9 seconds, a slightly elevated aspartate aminotransferase (AST) of 40 mmol/L, CRP of 150 mg/L, hypoalbuminemia of 2.4 g/dL, hypertriglyceridemia of 151 mg/dL, and ferritin above the maximum range value (>3,3511 ng/mL). A septic evaluation was initiated given her concerning clinical presentation, and her blood and urine cultures were both negative. Chest X-ray revealed no effusion or focal consolidations. Transthoracic echocardiogram showed normal systolic and diastolic function, no pericardial effusion, and no valvular abnormalities. Abdominal ultrasound was remarkable for hepatosplenomegaly. In consultation with the hospitalist service, a multidisciplinary team consisting of specialists in pediatric hematology/oncology, allergy/immunology, and pediatric rheumatology was formed to aid with the diagnostic evaluation and treatment plan.

## Discussion

The differential diagnosis of prolonged arthralgias, rash, and fevers should include viral, bacterial, or postinfectious arthritis, malignancy, and rheumatologic or autoinflammatory conditions. Multisystem inflammatory syndrome in children (MIS-C) was considered, although thought unlikely due to the lack of evidence of precedent or concurrent COVID-19 infection, pancytopenia, myocarditis, and significant gastrointestinal symptoms. Lyme serology had been completed previously and was negative. Malignancy was essentially ruled out with bone marrow biopsy revealing no blasts or other abnormal cell types but was significant for mildly hypercellular marrow and hemophagocytosis. Rheumatoid factor, cyclic citrullinated peptide (CCP), and human leukocyte antigen B27 (HLA-B27) were negative. Interleukin-2 (IL-2) receptor was strongly positive and markedly elevated greater than two standard deviations above the upper limit of normal at 2,766.6 pg/mL aiding in the final diagnosis.

Her laboratory trends continued to show a significant increase in ferritin; decreasing hemoglobin, platelets, fibrinogen, and ESR; and increasing AST/ALT and triglycerides. Along with her rheumatologic symptoms, her condition was concerning for MAS as a sequela of sJIA, as she met the criteria for the diagnosis of hemophagocytic lymphohistiocytosis (HLH). Her triglycerides peaked at 232 mg/dL, while fibrinogen and platelets decreased to 168 mg/dL and 181 × 10^3^/uL, respectively. This decreasing fibrinogen also correlated with a diminishing sedimentation rate as expected. Her transaminitis worsened with AST and ALT being 97 mmol/L and 66 mmol/L, respectively. Based on the diagnostic criteria, along with those of systemic juvenile idiopathic arthritis, the diagnosis of macrophage activation syndrome (MAS) was made and agreed upon by our subspecialty teams.

Macrophage activation syndrome (MAS) is a rare but life-threatening complication of systemic inflammatory syndromes and is most notably associated with sJIA [1,2]. The pathophysiology is related to a dysfunctional immune response involving activated T lymphocytes and hemophagocytic macrophages, resulting in the excess secretion of pro-inflammatory cytokines [1-3]. Macrophage activation syndrome is considered a secondary hemophagocytic lymphohistiocytosis (HLH) since it is associated with autoimmune disease [4]. While MAS can be the initial presentation of sJIA, it can also mimic a flare of sJIA, often making the diagnosis delayed and difficult to make [5]. The most notable clinical features of MAS include unremitting fever, hepatosplenomegaly, lymphadenopathy, central nervous system (CNS) dysfunction, and hemorrhagic symptoms in the most severe form of the disease [2,3]. Laboratory evidence includes cytopenias, coagulopathies, hypoalbuminemia, low fibrinogen, elevated ferritin, transaminitis, hypertriglyceridemia, and markedly elevated IL-2 receptor [2,3]. Bone marrow biopsy is significant for hemophagocytic activity [2]. In one study, the most useful indicators of MAS included hemorrhage, CNS dysfunction, decreased platelet count, increased AST, leukopenia, and decreased fibrinogen [6]. Research has found that a drop in certain laboratory values can be the most useful in identifying MAS, including WBC count, platelet count, and fibrinogen levels [3,4]. Although many still use the HLH-2004 diagnostic guidelines to diagnose MAS, Ravelli et al. determined the following 2016 criteria to classify MAS associated with known or suspected sJIA in a febrile patient: ferritin > 684 ng/mL and any two of the following: platelet count ≤ 181 × 10^9^/L, aspartate aminotransferase > 48 units/L, triglycerides > 156 mg/dL, and fibrinogen ≤ 360 mg/dL [6].

Prompt treatment is associated with more favorable outcomes, making early diagnosis extremely important [3,4,7]. Initial treatment is the administration of high-dose corticosteroids, usually starting with intravenous methylprednisolone for 1-3 days followed by prolonged taper and transition to oral therapy [4]. Cyclosporine A is another option for those nonresponders to corticosteroids [4]. One study found success with etanercept in those who did not respond to corticosteroids or cyclosporine A [3]. Another study found that the addition of the IL-1 receptor antagonist anakinra to traditional immunosuppressive therapies was successful in treating those with severe MAS [7].

The patient was started on 1 g of methylprednisolone daily for a three-day course while inpatient. She was initiated on daily treatment with the IL-1 receptor antagonist anakinra at a dose of 100 mg. Rapid significant clinical improvement was seen with initiation of therapy along with gradual improvement in biomarkers with normalizing triglycerides, ferritin, and liver enzymes. After the completion of intravenous steroids, she was transitioned to oral prednisone 60 mg daily with a planned 5 mg taper every other day until a 30 mg maintenance dose was reached. Unfortunately, she has not been able to wean lower than 30 mg daily even on anakinra. Like many patients with sJIA, her clinical course has waxed and waned, and alternative steroid-sparing agents will be pursued for her care.

## Conclusions

Overall, strong consideration for HLH/MAS should be considered in any pediatric patient presenting with fever of unknown origin and evidence of systemic inflammatory response, especially when an infectious etiology has been ruled out. Various diagnostic criteria exist but most involve elevated ferritin, triglycerides, transaminases, cytopenias, and evidence of hemophagocytosis in the bone marrow. IL-2 receptor assay greater than two standard deviations above normal is a more specific marker that can be obtained. Prompt diagnosis leads to improved clinical outcomes due to the early initiation of treatment. That treatment consists of a burst of high-dose corticosteroids for three days followed by a prolonged taper. Other adjunctive treatment includes the IL-1 receptor antagonist anakinra or other biologic therapies.
